# Three-day antibiotic duration in patients with pneumonia: A sixty-eight–hospital cohort

**DOI:** 10.1017/ash.2023.241

**Published:** 2023-09-29

**Authors:** Valerie Vaughn, Lindsay Petty, David Ratz, Elizabeth McLaughlin, Tawny Czilok, Jennifer Horowitz, Anurag Malani, Danielle Osterholzer, Scott Flanders, Tejal Gandhi

## Abstract

**Background:** Since 2019, community-acquired pneumonia (CAP) guidelines have recommended hospitalized patients be treated until clinical “stability and for no less than 5 days.” However, randomized trials have reported that, in patients who stabilize by hospital day 3, very short antibiotic durations (eg, 3 days) are noninferior to longer durations. How these trial results relate to real-world practice is unknown. **Methods:** Using a 68-hospital cohort study of hospitalized, general-care adults with CAP, we aimed to (1) quantify the percentage of patients who—according to trial criteria—qualify for a 3-day antibiotic duration, (2) quantify the percentage who actually received a 3-day duration, and (3) assess 30-day outcomes. Patients were considered to have CAP if they had a pneumonia discharge diagnosis and met clinical criteria for CAP. Patients with concomitant infections (including COVID-19), admission to intensive care, or severe immunocompromise were not included. **Results:** Between February 23, 2017, and August 3, 2022, 36,064 patients with CAP were included. Of those, 48.2% (9,826 of 36,064) were excluded due to a condition or organism ineligible for the 3-day treatment (Fig. 1). Of the 18,690 patients remaining, 52.6% (9,826) were unstable on day 3 and thus were ineligible for the 3-day treatment. Therefore, of all 36,064 patients, only 8,864 (24.6%) would be eligible under trial criteria for a 3-day treatment. Notably, 5,493 (55.9%) of 9,826 patients unstable on day 3 would be eligible for 5 days of treatment under national guidelines. In practice, use of 3–4-day treatment was rare, occurring in 599 (6.8%) of 8,864 patients eligible for a 3-day treatment versus 660 (6.7%) of 9,826 patients unstable on day 3 (*P* = .945). Use of 3–4-day treatment increased over time and comorbidities that could mimic CAP or a negative procalcitonin were more common in patients who received a 3–4-day treatment whereas specific symptoms of CAP were less common (Fig. 2). After adjustments, patients eligible for a 3-day duration who received a 3–4 day treatment versus a ≥5-day treatment had higher 30-day mortality (aOR, 1.87; 95% CI, 1.32–2.64) and readmission (aOR, 1.35; 95% CI, 1.17–1.56). **Conclusions:** Across 68 hospitals, <25% of patients hospitalized with CAP would be eligible for a 3-day antibiotic treatment. Though increasing over time, there was little use of 3–4-day treatments and, when prescribed, outcomes were worse, potentially due to CAP misdiagnosis. Given the small number of patients eligible for 3-day treatment, and the potential harm with too-short durations, it may be prudent to focus on increasing the use of 5-day treatments.

**Figure:**
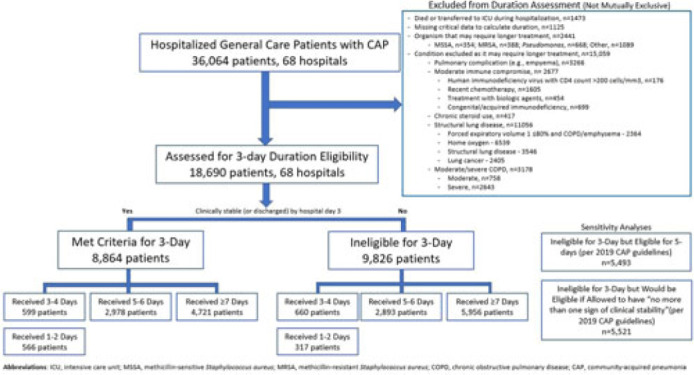

**Disclosures:** None

